# Supplementary material for: Research on optimization of perforation parameters for formation fractures based on response surface optimization method
Source: PLoS One. 2021 Aug 18;16(8):e0255793. doi: 10.1371/journal.pone.0255793 (PMC8372901; doi:10.1371/journal.pone.0255793)
Supplement: Attachment — Submitted filename: Response to Reviewers.docx [file pone.0255793.s001.docx]

**Response to Editor：**

Dear Editor,

Thank you very much for your work on my paper.

I have further revised the paper according to the reviewers' suggestions.

Sincerely Yours,

Wei Liu

2021/07/16

**Response to Reviewer #1:**

Dear professor,

Thank you for your comments to this paper. Now reply to your questions as follows.

Q1: In Section " Unit and simulation method validation"

Figure 3(b) should mark cluster 1, cluster 2 and cluster 3. Otherwise, reader cannot be clearly recognized.

A1: Figure 3(b) has been modified and cluster 1, cluster 2 , cluster 3 has been marked. Please see Figure 3(b) for details. Thank you.

Q2: In Section "Calculation model"

Figure 5 should mark the setting part of pipe flow unit and connection unit. Otherwise, the relationship between pipe flow unit and connection unit mentioned in section "Material AND Methods" setting model is not clear.

A2: Yes, you are right. If i can’t mark the setting part of pipe flow unit and connection unit, the reader may be not clear the relationship between pipe flow unit and connection unit mentioned. According to your opinion, Figure 5 has been modified. Please see Figure 5 for details.Thank you.

Q3: In Section "Response optimization design method"

The response surface optimization method process in Figure 6 shows that the response surface update and response surface based optimization should be sequential, not parallel, Please amend.

A3: Figure 6 has been modified. Please see Figure 6 for details.Thank you.

Q4: In Section "Response surface optimization design scheme"

The differences between BBD and CCD methods as well as the central point were introduced. The central, cubic, and axial points of BBD and CCD should be described.

A4: The author added a comparative description of BBD and CCD methods, and also added a different comparison diagram of BBD and CCD, which is shown in Figure 7 in the updated paper.Thank you .

Q5: In Section "Model construction and test"

Why the equation based on response surface optimization can optimize the parameters under the global response. In what ways does the equation prove to be more predictive? Please specify.

A5: First, response surface optimization (RSM) is an optimization method that integrates experimental design and mathematical modeling. By conducting tests on representative local points, the function relationship between factors and results in the global scope is regressed and fitted, and the optimal level value of each factor is obtained.The response surface optimization method in this paper is based on multiple linear regression equation, and 17 groups of test data are used for response regression analysis. Therefore, the optimization of perforation parameters in the global response can be completed.

Second, to explain the equilibrium equation and have better prediction of perforation parameters,The value of the multivariate phase relation can reflect the accuracy of the fitting equation. If the correlation coefficient R-squared is approaching 1, it indicates that the response has a strong correlation. As a result of the experimental design scheme, the fitting correlation coefficient R-squared is 0.94, which is close to 1 in height, proving the accuracy of the fitting equation. At the same time, in table parameter changes before and after optimization, the prediction deviations of the fracture cluster length developed by the perforation parameters before and after optimization were calculated by using the response equation. The prediction deviations of the equation before and after optimization were 1.2% and 0.4%, respectively, indicating that the response optimization equation has good predictability.

**Thank you for your comments to this paper again.**

Sincerely Yours,

Wei Liu

2021/07/16

**Response to Reviewer #2:**

Dear professor,

Thank you for your comments to this paper. Now reply to your questions as follows.

Q1: In Section "Unit and simulation method validation"

In Figure 4, there is little difference between the numerical simulation curve and the reference curve, so it is impossible to see at a glance whether the numerical simulation curve and the reference curve can be beautified to form a clearer expression. Please amend.

A1: Figure 4 has been modified. Please see Figure 4 for details.Thank you.

Q2: In Section " Simulation verification"

Figure12. Height difference between clusters is not clearly marked. Can the height difference between clusters be expressed in a better way? Please amend.

A2: Figure 12 has been modified. Please see Figure 12 for details.Thank you.

Q3: For the full text of the picture, the font size in the picture should be unified, there are some big and some small. Please amend.

A3: The text size of the pictures in the whole text has been adjusted uniformly. Please see all picture for details.Thank you.

Q4: The font of the full text table and the picture as well as the table should be unified. Some fonts in the picture and the table are not the same.Please amend.

A4: The tables and pictures in the full text have been modified. See the tables and pictures in the revised draft for details.Thank you.

Q5: In Section "Reference"

Some journal titles, such as Ref. 18, should be capitalized. Please amend.

A5: References have been modified as a whole, please refer to the references in the revised text for details.Thank you.

**Thank you for your comments to this paper again.**

Sincerely Yours,

Wei Liu

2021/07/16
